# A Novel High-Content Screening-Based Method for Anti-*Trypanosoma cruzi* Drug Discovery Using Human-Induced Pluripotent Stem Cell-Derived Cardiomyocytes

**DOI:** 10.1155/2021/2642807

**Published:** 2021-08-11

**Authors:** Diogo Crispim Nascimento Portella, Erik Aranha Rossi, Bruno Diaz Paredes, Tanira Matutino Bastos, Cássio Santana Meira, Carolina Vasques Kymie Nonaka, Daniela Nascimento Silva, Alex Improta-Caria, Diogo Rodrigo Magalhaes Moreira, Ana Cristina Lima Leite, Gevanio Bezerra de Oliveira Filho, José Maria Barbosa Filho, Ricardo Ribeiro dos Santos, Milena Botelho Pereira Soares, Bruno Solano de Freita Souza

**Affiliations:** ^1^Gonçalo Moniz Institute, Oswaldo Cruz Foundation (FIOCRUZ), Salvador, Brazil; ^2^D'Or Institute for Research and Education (IDOR), Salvador, Brazil; ^3^Center for Biotechnology and Cell Therapy, São Rafael Hospital, Salvador, Brazil; ^4^SENAI Institute for Innovation in Advanced Health Systems, SENAI CIMATEC, Salvador, BA, Brazil; ^5^Post-Graduate Program in Medicine and Health, Faculty of Medicine, Federal University of Bahia, Bahia, Brazil; ^6^Department of Pharmaceutical Sciences, Federal University of Pernambuco, Recife, PE, Brazil; ^7^Federal University of Paraiba, João Pessoa, PB, Brazil; ^8^National Institute of Science and Technology for Regenerative Medicine, Rio de Janeiro, RJ, Brazil

## Abstract

Chagas disease is caused by *Trypanosoma cruzi* infection and remains a relevant cause of chronic heart failure in Latin America. The pharmacological arsenal for Chagas disease is limited, and the available anti-*T. cruzi* drugs are not effective when administered during the chronic phase. Cardiomyocytes derived from human-induced pluripotent stem cells (hiPSC-CMs) have the potential to accelerate the process of drug discovery for Chagas disease, through predictive preclinical assays in target human cells. Here, we aimed to establish a novel high-content screening- (HCS-) based method using hiPSC-CMs to simultaneously evaluate anti-*T. cruzi* activity and cardiotoxicity of chemical compounds. To provide proof-of-concept data, the reference drug benznidazole and three compounds with known anti-*T. cruzi* activity (a betulinic acid derivative named BA5 and two thiazolidinone compounds named GT5A and GT5B) were evaluated in the assay. hiPSC-CMs were infected with *T. cruzi* and incubated for 48 h with serial dilutions of the compounds for determination of EC50 and CC50 values. Automated multiparametric analyses were performed using an automated high-content imaging system. Sublethal toxicity measurements were evaluated through morphological measurements related to the integrity of the cytoskeleton by phalloidin staining, nuclear score by Hoechst 33342 staining, mitochondria score following MitoTracker staining, and quantification of NT-pro-BNP, a peptide released upon mechanical myocardial stress. The compounds showed EC_50_ values for anti-*T. cruzi* activity similar to those previously described for other cell types, and GT5B showed a pronounced trypanocidal activity in hiPSC-CMs. Sublethal changes in cytoskeletal and nucleus scores correlated with NT-pro-BNP levels in the culture supernatant. Mitochondrial score changes were associated with increased cytotoxicity. The assay was feasible and allowed rapid assessment of anti-*T. cruzi* action of the compounds, in addition to cardiotoxicity parameters. The utilization of hiPSC-CMs in the drug development workflow for Chagas disease may help in the identification of novel compounds.

## 1. Introduction

Chagas disease, caused by the hemoflagellate protozoan *Trypanosoma cruzi*, affects approximately 6 to 7 million people worldwide [1, 2]. During the chronic phase, cardiac involvement occurs in up to 30% of the cases, leading to chronic Chagas cardiomyopathy (CCC), a disease that continues to be ranked among the most frequent etiologies of chronic heart failure in Latin American countries [3, 4].

Currently, benznidazole and nifurtimox are the only medications available to treat Chagas disease, both with proven efficacy when administered during the acute phase of the disease, which is often underdiagnosed [2]. In addition, treatment with these drugs can lead to serious adverse effects in some patients [5]. Therefore, there is an urgent need to increase the therapeutic arsenal for chronic Chagas disease through drug discovery or repurposing [2, 6]. In this context, the incorporation of innovative approaches in the preclinical *in vitro* screening process of anti-*T. cruzi* may contribute to accelerate the drug discovery process.

In the past years, human-induced pluripotent stem cells (hiPSCs) have contributed to drug discovery and toxicological studies applied to cardiovascular diseases [7, 8]. hiPSCs can be differentiated into any adult cell type, including cardiomyocytes, thus representing an invaluable tool for cardiovascular research, disease modeling, cardiotoxicity screening, and drug discovery [9]. Cardiomyocyte infection and parasite persistence are key factors in the pathophysiology of Chagas heart disease [10]. Therefore, studies with cardiomyocytes produced from hiPSCs (hiPSC-CMs) hold the potential to advance current knowledge about the disease pathogenesis and accelerate drug discovery and development, by facilitating preclinical assessments of toxicity and efficacy in relevant human cells [11].

Unpredicted cardiotoxicity is one of the main causes of drug withdrawal from the market and is the result of the low predictive value of currently available methods for preclinical cardiac toxicity testing [12]. This can be partially attributed to significant interspecies genetic and functional differences critical to the cardiomyocytes, which may influence the results obtained from animal studies [13]. hiPSC-CM-based assays offer the possibility of simultaneous evaluation of antitrypanocidal activity and cardiotoxicity in human cells. In this study, we evaluated a novel *in vitro* drug discovery method using *T. cruzi-*infected hiPSC-CMs and multiparameter analyses using a high-content screening (HCS) platform.

## 2. Methods

### 2.1. Ethics Statement

Cell reprogramming and experiments with hiPSCs received approval from the Ethics and Research Committee (IRB) at São Rafael Hospital (CAAE 20032313.6.0000.0048).

### 2.2. hiPSC Culture

We used two hiPSC lines obtained from two donors, previously obtained by integration-free reprogramming of erythroblasts with episomal vectors [14]. The cells were plated in Matrigel-coated wells (Corning; New York, NY, USA) and cultured with mTeSR1™ (Stem Cell Technologies; Vancouver, Canada). The medium was exchanged daily, and the cells were passaged with ReleSR (Stem Cell Technologies; Vancouver, Canada) when 80% confluence was reached, followed by replating in a 1 : 10 split ratio.

### 2.3. Cardiomyocyte Differentiation

hiPSCs were differentiated into cardiomyocytes using the PSC Cardiomyocyte Differentiation Kit (Thermo Fisher Scientific; Waltham, MA, US). Briefly, the hiPSCs were dissociated with ReleSR (Stem Cell Technologies; Vancouver, Canada) into a single cell suspension and replated at a 1 : 8 ratio in 12-well Matrigel-coated wells, being cultured with mTeSR1 (Stem Cell Technologies; Vancouver, Canada). During the first 24 h after plating (Day -3), 10 *μ*M Y27632 (ROCK Inhibitor, STEMCELL Technologies; Vancouver, Canada) was added to the culture medium. On Days -2 and -1, the hiPSC medium was exchanged, and on Day 0, mTeSR1 was replaced by Cardiomyocyte Differentiation Medium A, followed by Cardiomyocyte Differentiation Medium B on Day 2 and Cardiomyocyte Maintenance Medium on Day 4. The medium was then renewed every two days with Cardiomyocyte Maintenance Medium. Spontaneous beating clusters began to be observed on protocol Day 8.

For immunofluorescence, the cells were fixed with 4% paraformaldehyde (PFA), washed with PBS, and permeabilized with 0.05% Triton solution. The following primary antibodies and dilutions were used, with overnight incubation at 4°C: sarcomeric alpha-actinin (Sigma-Aldrich, St. Louis, MI, USA; 1 : 100), MF20 (DSHB, Iowa City, IA; 1 : 100), anti-cTNT (Thermo Fisher Scientific; 1 : 100), and GATA-4 (Santa Cruz Biotechnology, Dallas, TX, USA; 1 : 100). The cells were incubated with the secondary antibodies, for 1 h at room temperature with anti-mouse IgG Alexa Fluor 568-conjugated or anti-rabbit IgG Alexa Fluor 488-conjugated, both diluted at 1 : 500 (Thermo Fisher Scientific). Images were captured using a confocal microscope (Fluoview 1000, Olympus, Tokyo, Japan).

### 2.4. RT-qPCR Analysis

RNA was extracted from the cells with TRIzol reagent (Invitrogen, Carlsbad, CA) and the concentration determined by photometric measurement. A High-Capacity cDNA Reverse Transcription Kit (Applied Biosystems, Foster City, CA, USA) was used to synthesize cDNA from 1 *μ*g of RNA, according to the manufacturer's recommendations. Synthesis of cDNA and RNA expression analysis was performed by Real-Time PCR using TaqMan Gene Expression Assay for *GATA4* (Hs 01034628_m1), *NKX2.5* (Hs 00231763_m1), and *TNNT2* (Hs 00943911_m1). All reactions were run in duplicate on an ABI 7500 Real-Time PCR System (Applied Biosystems) under standard thermal cycling conditions. A nontemplate control (NTC) and nonreverse transcription controls (No-RT) were also included. The samples were normalized with *GAPDH* (endogenous control). The threshold cycle (2 − ΔΔCt) method of comparative PCR was used to analyze the results [15]. Data was analyzed using GraphPad software version 9.

### 2.5. Flow Cytometry

The cells were dissociated into single-cell suspensions by incubation with trypsin-EDTA solution (Thermo Fisher Scientific; Waltham, MA, US), at 37°C for 5 min, followed by centrifugation at 350 g for 5 min at room temperature. The cells were counted and stained with an APC-conjugated anti-TRA1-60 antibody (BD Biosciences; Franklin Lakes, USA) for pluripotency evaluation. The efficiency of cardiac differentiation was evaluated by staining with anticardiac troponin T (Thermo Fisher Scientific; Waltham, MA, US), after permeabilization with 0.3% Triton X-100 solution (Sigma-Aldrich; St. Louis, MI, United States). The secondary antibody anti-mouse IgG conjugated with Alexa Fluor 647 was then used (Thermo Fisher Scientific; Waltham, MA, US). Data acquisition was performed with the LSR Fortessa flow cytometer (BD Biosciences; Franklin Lakes, USA) using the FACSDiva v.6.3 acquisition and analysis software.

### 2.6. *T. cruzi* Infection

Infection of cardiomyocytes was performed using the Y strain *T. cruzi*. Trypomastigotes were obtained from *T. cruzi*-infected BALB/c mice at 7 d.p.i., as previously described [16] and maintained *in vitro* by infecting LLC-MK2 cells (ATCC CCL-7). The Animal Ethics Committee at Gonçalo Moniz Institute, Fiocruz, approved this protocol under the number 17/2017. The cells were cultured in DMEM with 10% fetal bovine serum and 1% penicillin-streptomycin (10,000 U/mL) (Thermo Fisher Scientific; Waltham, MA, US) and incubated at 37°C and 5% CO_2_ for 7 days before harvesting of free trypomastigote forms in the supernatant.

*T. cruzi* infection experiments were performed with hiPSC-CMs obtained from three donors, two generated in our laboratory and the third one commercially obtained (Pluricell, São Paulo, Brazil). The characterization of commercially obtained hiPSC-CMs was previously published [17]. hiPSC-CMs (2 × 10^4^ cells/well) were plated in 96-well plates coated with Matrigel (Corning; New York, NY, US) and kept at 37°C and 5% CO_2_ for 24 h. Then, the cells were infected with 1 × 10^5^ or 2 × 10^5^ trypomastigotes/well (multiplicity of infection (MOI) 5 or 10, respectively). The assay was adapted to 384-well plates, using 7 × 10^3^ cells/well and infection with 3.5 × 10^4^ trypomastigotes/well (MOI 5) for 24 h. On the following day, the wells were washed, and fresh media was added with the selected compounds.

### 2.7. Compounds

Three compounds endowed with anti-T. cruzi activity previously determined in conventional assays were tested: a betulinic acid derivative named BA5 and two thiazolidinone compounds named GT5A and GT5B [18–22]. The compounds had a degree of purity > 95%, as previously described [20]. Stock solutions at 10 mM were prepared by dissolving the lyophilized compounds in dimethyl sulfoxide (DMSO, OriGen; Austin, TX, US). Benznidazole (Lafepe; Recife, PE, Brazil), a gold standard anti-*T. cruzi* compound, was used for comparison. Other molecules—doxorubicin and endothelin-1 (both from Sigma-Aldrich; St. Louis, MI, United States)—were used in the nonlethal toxicity standardization assay.

### 2.8. Pharmacological Assays and High-Content Imaging Analysis

hiPSC-CMs were plated in 96- or 384-well plates at densities of 2 × 10^4^ cells/well and 7 × 10^3^ cells/well, respectively, and infected for 24 h, as described in the previous section. After incubation with the compounds at different concentrations for 48 h, the cells were fixed with 4% PFA and labeled with Hoechst 33342 (Thermo Fisher Scientific; Waltham, MA, US) or DRAQ5 (eBioscience; Santa Clara, CA, US). The images were acquired with the Operetta High Content System (PerkinElmer; Waltham, MA, US). Nuclei were delimited by the Hoechst 33342 or DRAQ5 (segmentation) channel using the Harmony software. The cytoplasm was segmented through the Alexa 594 channel, corresponding to the troponin T labeling. Intracellular amastigotes were detected as spots stained with DRAQ5 in the cytoplasm. Mock-infected control cells were used to exclude other nonspecific cytoplasmic spots from the analysis, by using morphological and fluorescence intensity parameters for selection. The experiments were carried out in triplicate for each test condition. Following cell segmentation and parameter selection, the total number of cells, the number of infected cells, the total number of amastigotes, the number of amastigotes per cell, and the infection rate were calculated. The cytotoxic concentration for 50% of the cardiomyocyte population (CC_50_), the inhibitory concentration for 50% of the amastigote population (EC_50_), and the selectivity index were calculated for test compounds and standard drug (benznidazole). The calculation of nonlinear regression to obtain the EC_50_ value was evaluated using Prism 7.04 (GraphPad Software; San Diego, CA, US).

The cytotoxicity of the compounds was evaluated by counting the number of cells in each well (lethal toxicity) and by evaluating other parameters of nonlethal toxicity: cell and nuclear morphology, damage to the cytoskeleton using Phalloidin-488 staining, and mitochondria biomass using MitoTracker Red staining (both from Thermo Fisher Scientific; Waltham, MA, EUA). The parameters were plotted in a principal component panel, where the drugs are clustered, and it was observed what groups were closer to control groups, after 48 hours of treatment. The parameters used in the evaluation of the cytoskeleton score were area, roundness, fluorescent intensity, and coefficient of variation (CV) of intensity to assess homogeneity of markers in the cell. To evaluate mitochondria biomass, we used fluorescent intensity, CV of intensity, the texture index SER “Hole” and texture index SER “Saddle,” and the texture-based analysis, to access the pixel intensity, showing the effects of the drugs on mitochondrial morphology (Figure S1).

### 2.9. NT-Pro-BNP Measurements

To evaluate cardiomyocyte mechanical stress and hypertrophy processes, the concentrations of the N-terminal prohormone of brain natriuretic peptide (NT-pro-BNP) in the cardiomyocyte culture supernatants were evaluated. Culture supernatants were collected 48 hours after incubation with the compounds, pooled, and frozen at -80°C until analysis with the commercially available kit Vidas® NT-pro-BNP (Biomerieux, Marcy, France), following the manufacturer's recommendations.

### 2.10. Statistical Analyses

Parametric data were evaluated using Student's *t*-test. Nonparametric data were assessed using the Mann–Whitney test. For comparison between three or more groups, the ANOVA test with Tukey's posttest for parametric data and the Kruskal-Wallis with Dunn's posttest for nonparametric data were used. Values of *p* < 0.05 were considered statistically significant. The EC_50_ values were obtained through nonlinear regression analyses and the selectivity index by dividing the average of the CC_50_ values over EC_50_ (IS = CC_50_ ÷ EC_50_). Correlations between continuous variables were evaluated by the Pearson or Spearman coefficients.

## 3. Results

**Figure 1:**
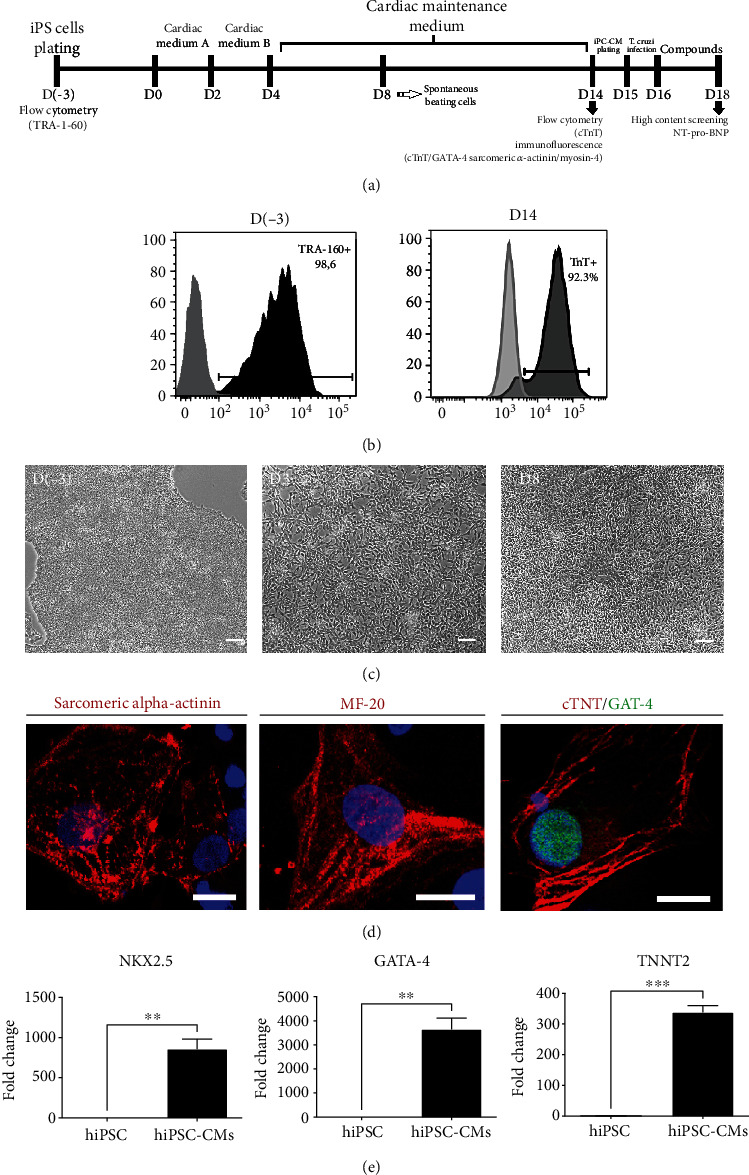
Cardiac differentiation induction in hiPSC and characterization of hiPSC-CMs. (a) Schematic experimental design. (b) Flow cytometry analysis of hiPSCs at D(-3), before plating for cardiac induction, for TRA-1-60 expression, and hiPSC-CM purity at differentiation D14 evaluated by cardiac troponin-T (cTNT) expression. Light grey histograms represent isotype controls. (c) Representative phase contrast micrographies of the different days postinduction of cardiac differentiation from hiPSCs. Bars = 50 *μ*m. (d) Representative confocal microscopy images of hiPSC-CMs stained with sarcomeric alpha-actinin, sarcomeric myosin (MF-20), cardiac troponin T (cTNT), and GATA-4 (all in red). Nuclei were stained with DAPI (blue). Bars = 20 *μ*m. (e) Gene expression analysis by RT-qPCR demonstrating mRNA expression of cardiac genes *NKX2.5*, *GATA-4*, and *TNNT2*, normalized to the levels of *GAPDH*. ^∗∗^*p* < 0.01; ^∗∗∗^*p* < 0.001.

**Figure 2:**
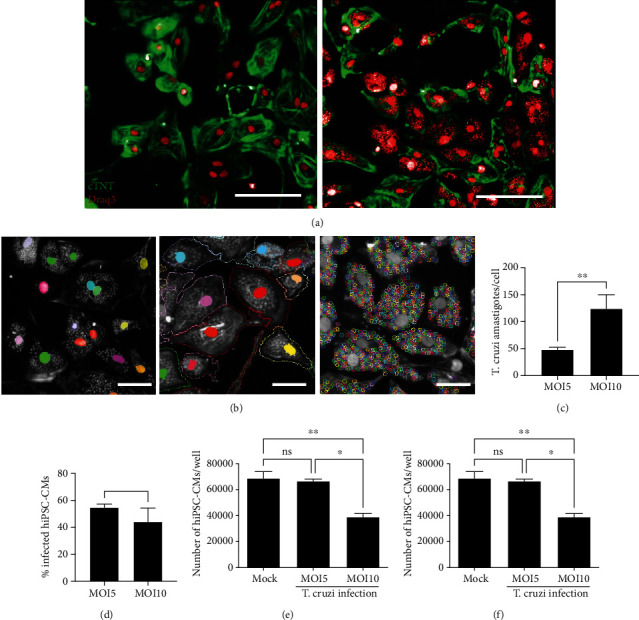
Infection of hiPSC-CMs with *T. cruzi* and high-content screening analysis. (a) Representative image of uninfected hiPSC-CM control, showing cTnT staining (green) and nuclei stained with Draq5 (red). (b) Representative image of hiPSC-CMs infected with *T. cruzi*, showing cTnT staining (green) nuclei and amastigotes stained with Draq5 (red). (c) Standardization of image analysis in Operetta High Content Imaging System, illustrating the steps of nuclei identification (left), followed by cytoplasm delimitation (middle), and *T. cruzi* amastigote spot identification (right). Quantification of the number of amastigotes/cell (d), percentage of infection (e), and number of hiPSC-CMs (f) 72 h following infection in MOI = 10 and MOI = 5. ^∗^*p* < 0.05; ^∗∗^*p* < 0.01; ns = not significant (*p* ≥ 0.05). Bars = 50 *μ*m.

Human iPSCs were induced to differentiate into hiPSC-CMs, generating a population of nearly 90% troponin T-positive beating cardiomyocytes at differentiation Day 14 (Figure 1). To evaluate the susceptibility of hiPSC-CMs to *T. cruzi* infection and to define the optimal MOI to be used in the assay, a preliminary test was performed using MOIs 5 and 10. hiPSC-CMs were highly permissive to *T. cruzi* infection, leading to similar infection rates (54% and 44% for MOIs 5 and 10, respectively). The average number of amastigotes per cell was significantly higher for MOI 10 compared to 5 (124 vs. 48, respectively) (Figure 2(a)). A nonstatistically significant tendency towards decreased hiPSC-CM numbers was found for MOI 10 (Figures 2(b) and 2(c)). MOI 5 was then selected for the following experiments.

**Figure 3:**
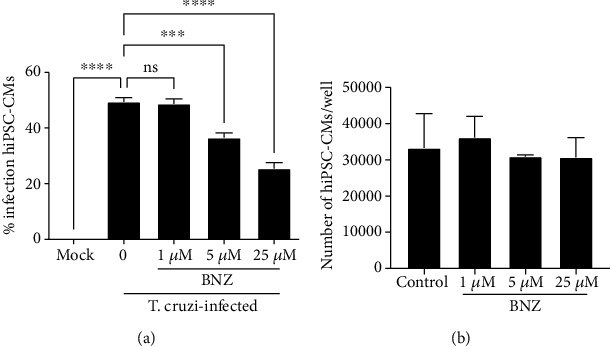
Effects of benznidazole (BNZ) on *T. cruzi*-infected hiPSC-CMs. (a) Percentage of infection BNZ-treated hiPSC-CMs. (b) Number of cardiomyocytes/well in cultures treated with BNZ and control. ^∗∗∗^*p* < 0.001; ^∗∗∗∗^*p* < 0.0001; ns = not significant (*p* ≥ 0.05).

Infected hiPSC-CMs were treated with the standard drug, benznidazole, in different concentrations. Benznidazole was effective in reducing the percentage of infection in a concentration-dependent manner (Figure 3(a)). None of the tested concentrations was associated with a reduction in the number of hiPSC-CMs, compared to untreated control cultures (Figure 3(b)).

Next, we used our hiPSC-CM-based infection model to evaluate the anti-*T. cruzi* activity of two synthetic and one semisynthetic compounds (GT5A, GT5B, and BA5), which were previously shown to exert potent anti-*T. cruzi* activities compared to the reference drug, benznidazole [22–24]. Among the compounds tested, benznidazole had the highest CC_50_ value, followed by GT5A, whereas compound GT5B had the lowest EC_50_ value (Table 1).

Nonlethal toxicity parameters were evaluated through morphological analysis of hiPSC-CMs incubated with the compounds in different concentrations, followed by staining with phalloidin, to evaluate the cytoskeleton/cell morphology, and MitoTracker, to evaluate mitochondria content/morphology. Nuclei morphology was evaluated by Hoechst 33342 staining. NT-pro-BNP levels in the culture supernatant were also measured. In the first step, hiPSC-CMs were incubated with the cardiotoxic drug doxorubicin or the hypertrophic molecule endothelin-1 (ET-1). Incubation with ET-1 was associated with signs of cell hypertrophy (Figures S2A and B), with increased intensity of phalloidin-labeled cytoskeleton compared to the untreated control group and to doxorubicin-treated cells, which presented reduced intensity of phalloidin fluorescence and cell size. These results correlated with the detection of high NT-pro-BNP levels in culture supernatants of hiPSC-CMs treated with ET-1 and a reduction after treatment with doxorubicin, which was associated with a significant increase in cell death (Figures S2A and B).

**Figure 4:**
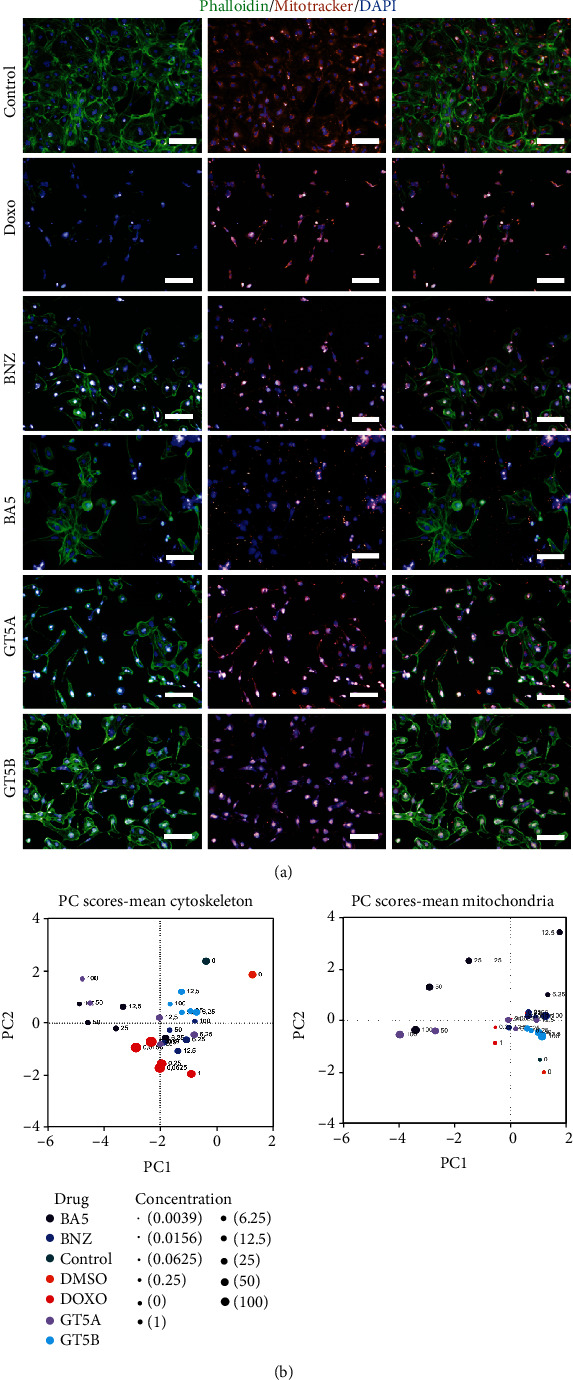
High-content imaging analysis of sublethal cardiotoxicity of anti-*T. cruzi* compounds. (a) Representative images of hiPSC-CMs untreated (control) or treated with 0.5% DMSO, 1 *μ*M doxorubicin (Doxo), benznidazole (BNZ), BA5, GT5A, or GT5B, all at the 25 *μ*M concentration. Cytoskeleton was stained with phalloidin (green) and mitochondria with MitoTracker (orange), and nuclei were stained with DAPI (blue). Bars = 100 *μ*m. (b) Principal component analysis demonstrating the effects of the compounds in different concentrations to the cytoskeleton and mitochondria scores.

Next, we evaluated the effects of the anti-*T. cruzi* compounds benznidazole, BA5, GT5A, and GT5B 48 hours following treatment in different concentrations (100, 50, 25, 12.5, and 6.25 *μ*M). Different parameters were combined, and principal component analyses were conducted, defining a cytoskeleton score and a mitochondrial score (Figure S1). While treatment with DMSO did not alter significantly either the cytoskeleton or the mitochondria scores compared to untreated controls, treatments with all compounds slightly altered these parameters, which generated a cluster for most of the concentrations tested (Figure 4). In addition to doxorubicin, treatment with GT5A in the concentrations of 50 and 100 *μ*M and BA5 in the concentrations of 12.5, 25, 50, and 100 *μ*M led to higher dispersion and distancing from the values obtained for untreated and DMSO-treated hiPSC-CMs (Figure 4).

**Figure 5:**
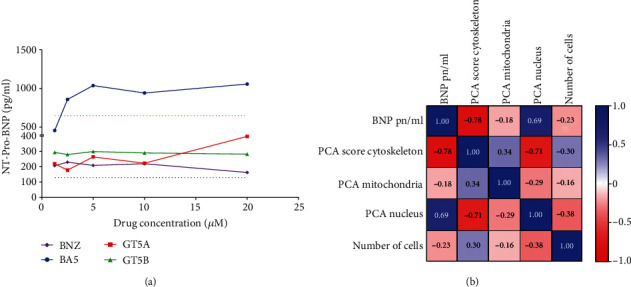
NT-pro-BNP analysis and correlations with morphology parameters. (a) NT-pro-BNP levels in the culture supernatant 48 h following treatment with the compounds in different concentrations. Dash lines represent NT-pro-BNP levels in the culture supernatant of untreated hiPSC-CMs (black) 48 h following treatment with endothelin-1 (orange). (b) Correlation matrix heat map demonstrating *R* values found for each comparison between the different variables.

Treatment with all compounds tested led to an increase in the secretion of NT-pro-BNP compared to untreated hiPSC-CMs, but a concentration-dependent increase was observed in BA5-treated cells, leading to NT-pro-BNP levels that, at the concentrations 2.5, 5, 10, and 20 *μ*M, surpassed the values observed after ET-1 stimulation (Figure 5(a)). Finally, we found that NT-pro-BNP levels in the culture supernatant showed a statistically significant correlation with the cytoskeleton score and nuclear morphology (Table 2 and Figure 5(b)).

**Figure 6:**
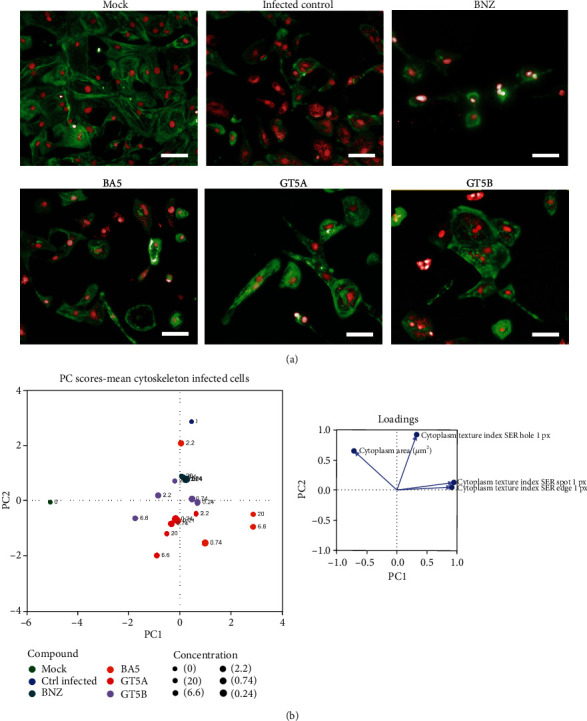
High-content imaging analysis of infected hiPSC-CMs treated with the compounds. (a) Representative images of hiPSC-CMs uninfected (mock) or infected with *T. cruzi* submitted to compound (BNZ (benznidazole), BA5, GT5A, or GT5B) testing at the concentration of 6.6 *μ*m. Bars = 50 *μ*m. (b) Principal component analysis demonstrating the effects of the compounds in different concentrations to the cytoskeleton-infected hiPSC-CM score.

To evaluate whether the high-content imaging strategy could also be applied to the study of compound effects in cell morphology of infected cells, we analyzed the experiments of *T. cruzi* infection generating a new score that combined measurements of spots, texture, and cytoskeleton staining (Figure 6). By principal component analysis, mock and *T. cruzi*-infected cells are displayed in separate regions of the plot, while the compounds brought the cell morphology parameters to an intermediate zone. Among the tested conditions, the cells treated with the compounds GT5B and GT5A were found to be more similar to mock-infected cells, while BA5 significantly altered cell morphology.

## 4. Discussion

hiPSC-CMs hold the potential to contribute to the anti-*T. cruzi* drug discovery process by increasing the predictive value of preclinical assays. Considering the need to develop new drugs for the treatment of Chagas disease, we established a multiparametric pharmacological assay for simultaneous evaluation of cardiotoxicity and anti-*T. cruzi* activity using hiPSC-CMs and a HCS platform with automated analysis, reducing bias and increasing confidence in the assay. Considering the role of parasite persistence in the myocardium, the preclinical confirmation that drugs with previously described antiparasitic actions (GT5A, GT5B, and BA5) are also safe and effective in cardiomyocytes brings valuable data for further development.

Treatment of infected hiPSC-CMs with the reference drug benznidazole resulted in an EC_50_ value of 5.9 *μ*M, which is situated within the range of EC_50_ values reported in the literature for benznidazole in other cell types [16, 18, 19]. Studies have shown that betulinic acid and its derivatives, such as BA5, inhibit the proliferation of epimastigotes and reduce the viability of trypomastigote forms [19, 20]. In our findings, BA5 had an EC_50_ of 3.2 *μ*M for reducing *T. cruzi* amastigotes in hiPSC-CMs, half of the EC_50_ value found for the reference drug, benznidazole. Previously, an EC_50_ value of 1.8 *μ*M was found for murine macrophages [20]. Thiazolidines are potent cruzain inhibitors and have been previously studied as anti-*T. cruzi* drugs [23]. Both GT5A and GT5B have been identified as potent agents with trypanocidal action with high selectivity index [24]. These compounds had EC_50_ values in hiPSC-CMs of 1.9 and 0.8 *μ*M values, respectively, much lower compared to benznidazole. A previous study in macrophages reported higher values of EC_50_ for GT5A and GT5B, with 4.2 and 2.9 *μ*M, respectively [24].

Our data also revealed cell type-specific toxicities for some of the compounds, which were not reported in previous studies with murine cells. BA5 showed lethal toxicity for hiPSC-CMs with a CC_50_ of 37 *μ*M, which is discrepant and significantly lower than the values previously reported for murine macrophages [22]. At lower concentrations, hiPSC-CMs demonstrated changes in the cytoskeleton score, suggesting cytoskeleton disorganization, along with high levels of NT-pro-BNP, a biomarker of myocardial stress. Interestingly, we demonstrated that the levels of NT-pro-BNP showed a statistically significant correlation with the cytoskeleton and nuclear scores, but not with the mitochondria score or lethal toxicity parameters.

The mechanical stability of the cardiomyocyte depends on the integrity of the cytoskeleton. Geometric changes in the cell membrane can lead to changes in electrophysiology [25]. Some drugs can induce cytoskeletal disorganization and mechanical stress or even stimulate a hypertrophic response. In the present study, we used phalloidin staining to assess the cytoskeleton, and using positive controls, we observed that this analysis can demonstrate, quantitatively, a hypertrophic response (assessed with treatment with ET-1) and disruption of the cytoskeleton (observed with the doxorubicin treatment). Interestingly, these findings correlated with increased levels of release of the NT-pro-BNP biomarker to the culture medium in the hypertrophic response induced by ET-1, while reduced levels were observed in the case of treatment with doxorubicin, which is in agreement with previous observations in the literature [26].

Dysfunctional mitochondria can compromise myocardial function [26, 27] as cardiomyocytes require high levels of ATP to function properly. Some drugs can be cardiotoxic by inducing mitochondrial damage, increasing oxidative stress, activating DNA damage response pathways, and increasing apoptosis [28]. By including mitochondria parameters in high-content analyses, the assay also allowed the identification of gross alterations in mitochondria that could lead to cardiotoxicity.

The compounds tested herein comprehend the two main classes of compounds currently explored as antiparasitic agents for Chagas disease: molecules with exclusive antiparasitic activity (GT5A and GT5B) and molecules with dual antiparasitic and anti-inflammatory/immunomodulatory activity (BA5). Another clinical significance of the compounds is their pharmacological profile. In cardiomyocytes, we found that all three compounds have IC50 values lower than the reference drug benznidazole, making them candidates for further development. However, the toxicity analyses showed that BA5 may present a cardiotoxicity profile, an observation similar to a high concentration of GT5A. But unlike GT5A, GT5B was not only the most potent compound in terms of anti-*T. cruzi* activity but also less toxic to hiPSC-CM, with less altered cell morphology, as demonstrated by multiparametric HCS analysis.

A limitation of the present study involves the degree of maturity of hiPSC-CMs. So far, hiPSC-CMs that have been used for the cardiotoxicity test show a structural phenotype compatible with fetal cardiomyocytes. The stage of development of hiPSC-CMs used in this study is in accordance with the literature, which reports that cardiac differentiation from hiPSC routinely leads to the generation of cells with an immature structural and functional phenotype, of the fetal type [29]. However, the impact that the hiPSC-CMs' maturation status has on the reaction capacity to the compounds is not clear [28, 29]. Considering that the degree of maturation of hiPSC-CMs can be a critical factor for obtaining more predictive tests, further studies should be carried out to optimize the process of obtaining these cells. There are already reports in the literature of methods for inducing maturation, including through the modulation of mechanical strength (afterload) or by culturing hiPSC-CMs in 3D [30, 31], which could be evaluated in future studies. Doxorubicin binds to cardiolipin and inhibits the respiratory chain and the depolarization of the membrane potential, among other mechanisms [32]. Mitochondria are involved in several cardiomyocyte functions, including fatty acid metabolism, amino acids, and ATP generation [33].

## 5. Conclusions

The use of hiPSC-CMs in the drug development workflow for Chagas disease has the potential to assist in the identification of new compounds and to predict cardiotoxicity. In this work, we established the test for infection and screening compounds with anti-*T. cruzi* activity in hiPSC-CMs, using multiparametric analyses on a high-content screening platform. This assay was able to confirm the anti-*T. cruzi* activity of BA5, GT5A, and GT5B and identified the compound GT5B as promising, due to its potency and low toxicity in cardiomyocytes. In addition to having the advantage of being based on the use of human cardiac cells affected by Chagas disease, the assay has the advantage of allowing the rapid assessment of anti-*T. cruzi* actions in addition to parameters of lethal and sublethal cardiotoxicity, which can increase the predictive value of the tests.

## Figures and Tables

**Table 1:** Cytotoxicity against hiPSC-CMs and anti-*T. cruzi* activity against intracellular amastigotes.

Compound	CC_50_ (*μ*M)	EC_50_ (*μ*M)	SI
BA5	37 ± (1.9)	3.2 ± (0.8)	12
GT5A	87 ± (22)	1.9 ± (0.6)	46
GT5B	27 ± (3.5)	0.8 ± (0.2)	33
Benznidazole	>100	5.9 ± (0.5)	>17

CC_50_: cytotoxicity concentration 50%; EC_50_: effective concentration at 50% inhibitory concentration for inhibition of *T. cruzi* amastigotes. Values are means ± SD of three independent experiments.

**Table 2:** Correlations between NT-pro-BNP levels and morphological parameters.

	PCA cytoskeleton score	PCA mitochondria score	PCA nucleus morphology	Number of cells
NT-pro-BNP levels	*p* = 0.006 *R* = −0.74	*p* = 0.792 *R* = −0.09	*p* = 0.027 *R* = 0.63	*p* = 0.473 *R* = −0.23

## Data Availability

Data is available upon reasonable request.

## Supplementary Materials

Supplementary Materials Figure S1: vectors included in the principal component analysis for cytoskeleton and mitochondria scores. Figure S2: morphology and NT-pro-BNP levels. (A) Representative images of hiPSC-CMs stained with phalloidin (green) and DAPI (blue) untreated (control) or 48 h following treatment with endothelin-1 (ET-1) or doxorubicin (Doxo). Bars = 50 *μ*m. (B) Quantification of phalloidin fluorescence intensity, number of hiPSC-CMs, and levels of NT-pro-BNP in the supernatant. ^∗∗^*p* < 0.01; ^∗∗∗^*p* < 0.001; ^∗∗∗∗^*p* < 0.0001; ns = not significant (*p* ≥ 0.05).
