# Supplementary material for: A Novel High-Content Screening-Based Method for Anti-Trypanosoma cruzi Drug Discovery Using Human-Induced Pluripotent Stem Cell-Derived Cardiomyocytes
Source: Stem Cells Int. 2021 Aug 11;2021:2642807. doi: 10.1155/2021/2642807 (PMC8380504; doi:10.1155/2021/2642807)
Supplement: Supplementary Materials — Figure S1: vectors included in the principal component analysis for cytoskeleton and mitochondria scores. Figure S2: morphology and NT-pro-BNP levels. (A) Representative images of hiPSC-CMs stained with phalloidin (green) and DAPI (blue) untreated (control) or 48 h following treatment with endothelin-1 (ET-1) or doxorubicin (Doxo). Bars = 50 μm. (B) Quantification of phalloidin fluorescence intensity, number of hiPSC-CMs, and levels of NT-pro-BNP in the supernatant. ∗∗p < 0.01; ∗∗∗p < 0.001; ∗∗∗∗p < 0.0001; ns = not significant (p ≥ 0.05). [file 2642807.f1.docx]

**Supplementary Figures**

**Figure S1. Vectors included in the principal component analysis for cytoskeleton and mitochondria scores.**

**Control**

**A**

**B**


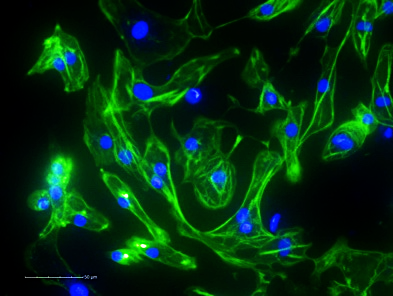

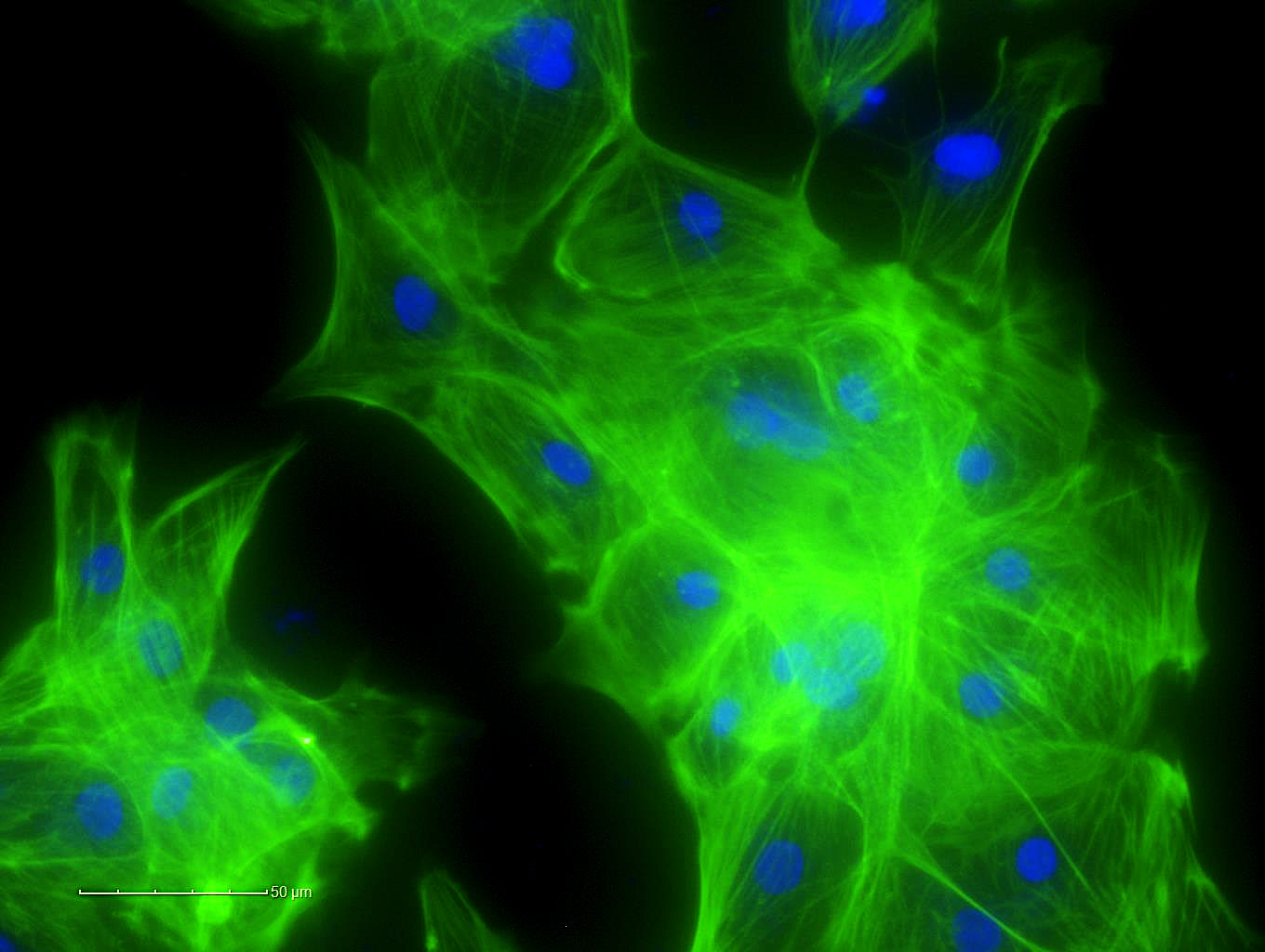

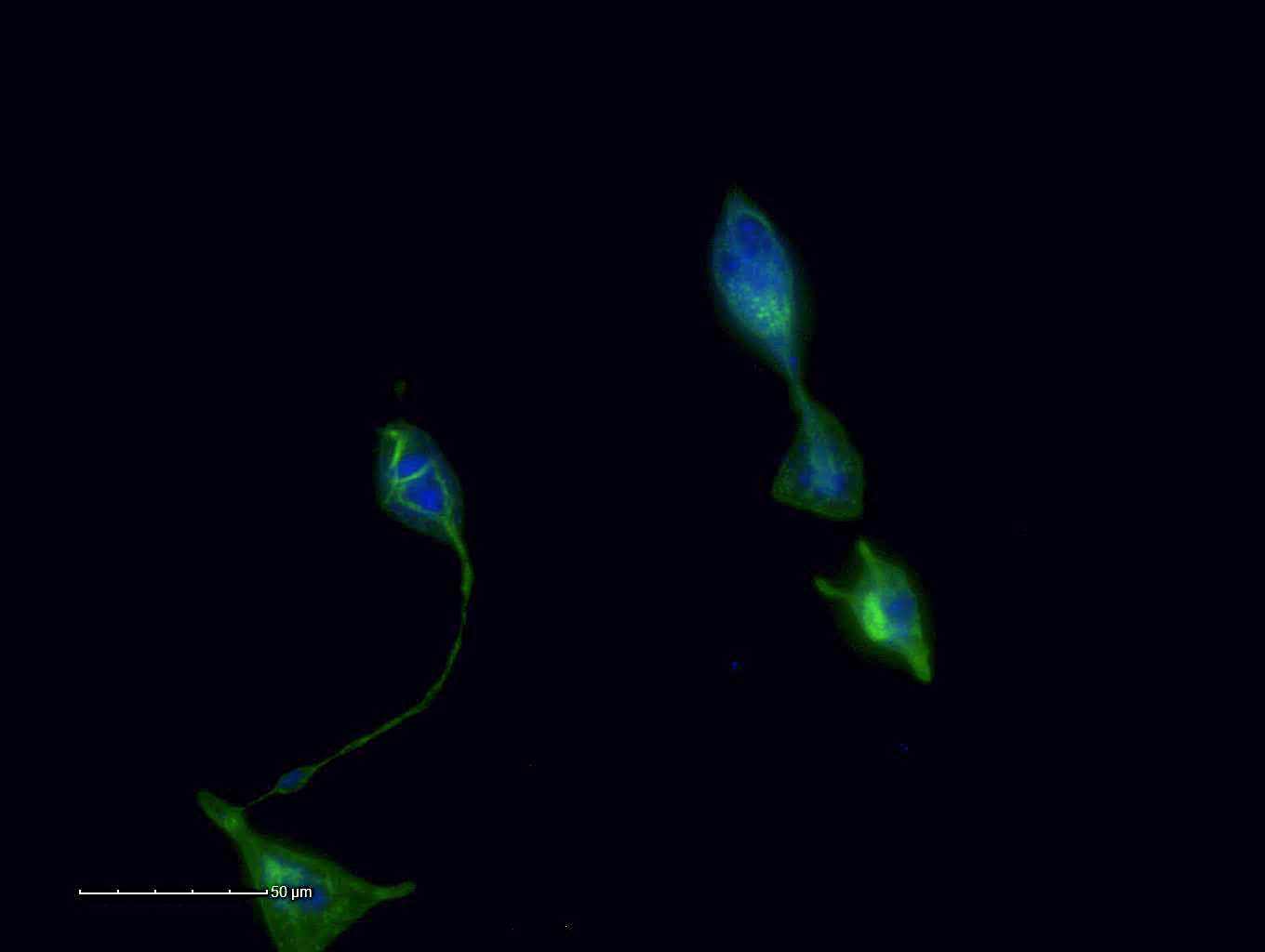


**ET-1**

**Doxo**

**C**

**Figure S2. Morphology and NT-Pro-BNP levels.** (A) Representative images of hiPSC-CMs stained with phalloidin (green) and DAPI (blue) untreated (control) or 48 h following treatment with endothelin-1 (ET-1) or doxorubicin (Doxo). Bars = 50 µm. (B) Quantification of phalloidin fluorescence intensity, number of hiPSC-CMs and levels of NT-Pro-BNP in the supernatant. **p <0.01; ***p<0.001; ****p<0.0001; ns = not significant (p≥0.05).
